# Development of short hairpin RNA expression vectors targeting the internal ribosomal entry site of the classical swine fever virus genomic RNA

**DOI:** 10.1186/s12896-023-00805-6

**Published:** 2023-09-08

**Authors:** Riai Okamoto, Nobumasa Ito, Yutaro Ide, Bouchra Kitab, Yoshihiro Sakoda, Kyoko Tsukiyama-Kohara

**Affiliations:** 1 Transboundary Animal Disease Center, Joint Faculty of Veterinary Medicine, Kagoshima University, Kagoshima, 890-0065 Japan; 2 Department of Disease Control, Faculty of Veterinary Medicine, Hokkaido University, Hokkaido, 060-0818 Japan; 3 Laboratory of Animal Hygiene, Joint Faculty of Veterinary Medicine, Kagoshima University, Kagoshima, 890-0065 Japan

**Keywords:** Classical swine fever virus, Internal ribosomal entry site, Domain III, siRNA, shRNA

## Abstract

**Background:**

Classical swine fever (CSF) is a fatal contagious disease affecting pigs caused by classical swine fever virus (CSFV). The disease can be transmitted by pigs and wild boars, and it is difficult to prevent and control. To obtain necessary information to establish the CSFV resistant animals in a future study, we designed lentiviral vector-delivered short hairpin RNAs (shRNAs) targeting the conserved domain III of the internal ribosomal entry site (IRES) of the CSFV genomic RNA.

**Results:**

First, we confirmed the effects of siRNAs on CSFV-IRES activity. We observed significant inhibition of CSFV-IRES activity by si42 (domain IIIa), si107 (domain IIIc), and si198 (domain IIIf) in SK-L cells and si56 (domain IIIb), si142 (domain IIId_1_) and si198 in HEK293 cells without affecting the amount of luciferase RNA. Next, we constructed lentiviral vectors expressing shRNA based on siRNA sequences. Treatment with shRNA-expressing lentivirus was examined at 7 and 14 days post infection in SK-L cells and HEK293 cells, and CSFV-IRES was significantly suppressed at 14 days (sh42) post infection in HEK293 cells without significant cytotoxicity. Next, we examined the silencing effect of siRNA on CSFV replicon RNA and observed a significant effect by si198 after 2 days of treatment and by shRNA-expressing lentivirus (sh56, sh142, and sh198) infection after 14 days of treatment. Treatment of sh198-expressing lentivirus significantly suppressed CSFV infection at 3 days after infection.

**Conclusion:**

The IRES targeting sh198 expressing lentivirus vector can be a candidate tool for CSFV infection control.

**Supplementary Information:**

The online version contains supplementary material available at 10.1186/s12896-023-00805-6.

## Introduction

Classical swine fever virus (CSFV) belongs to the genus *Pestivirus* and the family *Flaviviridae*. The virus possesses a single-stranded RNA genome with positive polarity that encodes structural (C, E^rns^, E1, and E2) and non-structural (N^pro^, p7, NS2, NS3, NS4A, NS4B, NS5A, and NS5B) polyproteins [1]. The 5’-untranslated region (UTR) contains an internal ribosomal entry site (IRES) in CSFV-RNA [2], similar to that in other pestiviruses, bovine viral diarrhea virus (BVDV) [3], and border disease viruses [4, 5].

Classical swine fever (CSF), which is caused by CSFV, emerged more than 200 years ago and continues to threaten the swine industry in Asian and South American countries [6, 7]. Recently, outbreaks were reported in Brazil, Columbia, Russia, Korea, and Japan [8, 9]. Transmission of CSFV is mediated by wild animals such as boars, and its prevention and control are challenging in most countries. Therefore, the CSFV epidemic remains uncontrolled to date.

An efficient CSFV vaccine was developed for CSF prevention [10]. However, the vaccine alone is insufficient to control CSFV infection, as mutations generated during epidemics in wild boars decrease the efficacy of the vaccine. A plausible approach is to generate CSFV infection-resistant animals that express shRNA targeting mutant CSFV, as shRNA can be designed to immediately adapt to mutant viruses. Genetically modified pigs exhibit resistance to CSFV infection [11, 12]. In addition, RNA interference inhibits CSFV replication [13].

To develop antivirals and CSFV-resistant animals, we designed siRNA- and shRNA-targeting domain III within the IRES region, which is highly conserved and important for CSFV replication [14].

## Results

### Design of siRNA targeting CSFV-IRES domain III region

The CSFV-IRES-expressing cells were established using the pCAG vector and swine SK-L and human HEK293 (pCI5) cells (Additional file 1: Fig. S1) as previously described [15]. The CSFV-IRES activity was measured as the ratio of firefly luciferase (F-luc) to renilla luciferase (RL) activity, as described in the Methods section.

**Fig. 1:**
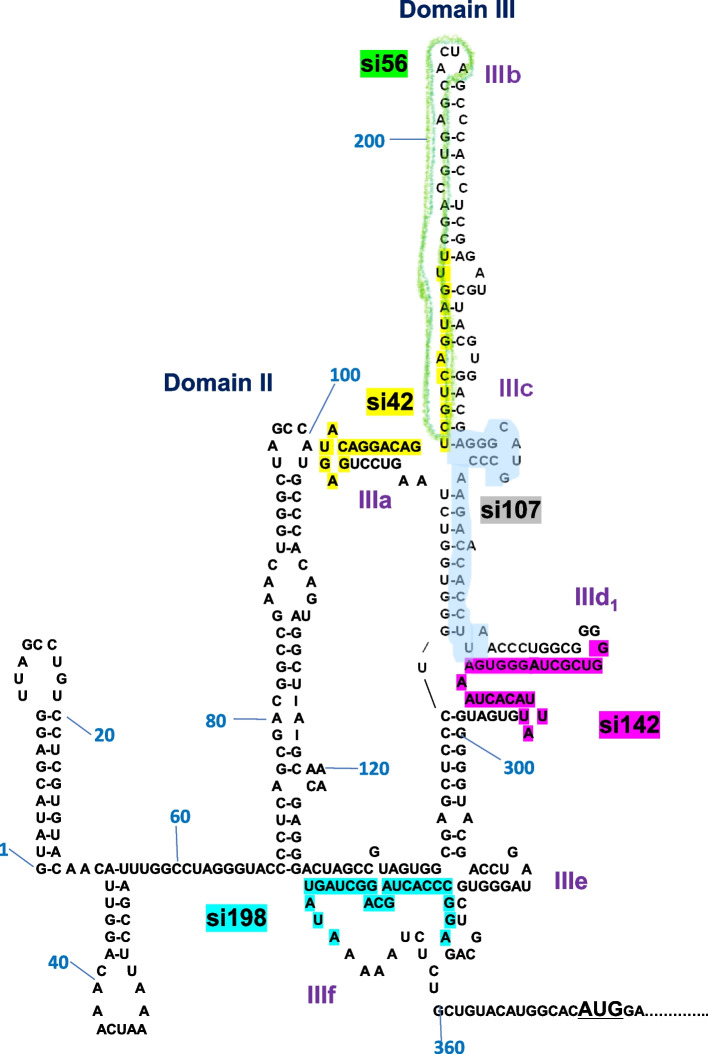
Position of siRNAs in domain III of CSFV-IRES. The positions of siRNA, si42, si56, si107, si142, and si198 in CSFV-IRES are indicated as dotted lines. Subdomains IIIa–IIIf are also indicated

The siRNAs were designed to target domain III of CSFV IRES, as described in the Methods section (Table 1). This is because domain III is highly conserved and is important for IRES function [16, 17] (Fig. 1).

**Table 1:** List of siRNAs targeting CSFV

Name	siRNA sequence
1. si42	5´-GAGUACAGGACAGUCGUCAGUAGUU-3´
2. si56	5′- CGUCAGUAGUUCGACGUGAGCACUA-3′
3. si107	5´-AGGGCAUGCCCAAGACACACCUUAA-3´
4. si142	5´-GGUCGCUAGGGUGAAAUCACAUUAU-3´
5. si198	5´-AGAGGCCCACUAGCAGGCUAGUAUA-3´

### Examination of siRNA effect in CSFV-IRES-expressing cells

**Fig. 2:**
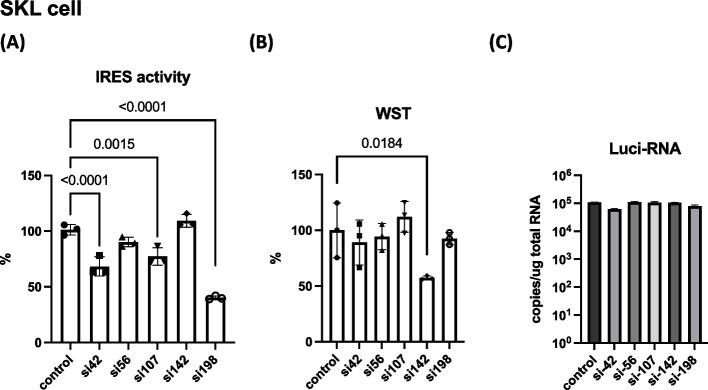
Comparison of siRNA with CSFV-IRES activity. **A** Percentage of IRES activity in control and siRNA-treated cells (72 h). One-way ANOVA and Dunnett’s multiple comparison test were conducted for all samples. *P*-value < 0.05 compared with control is indicated. **B** Cell viability was measured using the WST assay and indicated by the OD_450_ value. Vertical bars indicate the standard deviations. One-way ANOVA and Dunnett’s multiple comparison test were conducted for all samples. *P*-value < 0.05 is indicated. **C** Amount of luciferase RNA in siRNA-treated cells quantitated by qRT-PCR. Representative results of three experiments are shown

To evaluate the silencing effect of siRNA, we introduced si42, si56, si107, si142, and si198 into the CSFV-IRES-expressing SK-L cells [15] (Fig. 2). After 3 days, we found significant suppression of IRES activity by si42, si107, and si198 (Fig. 2A) without significant cytotoxicity (Fig. 2B). Cells treated with si142 exhibited significant cytotoxicity for unknown reasons. Luciferase RNA was measured by qRT-PCR as described in the Methods section, and similar amounts of RNA were detected in siRNA-treated cells (Fig. 2C).

### Construction of shRNA-expressing vectors targeting CSFV-IRES

**Fig. 3:**
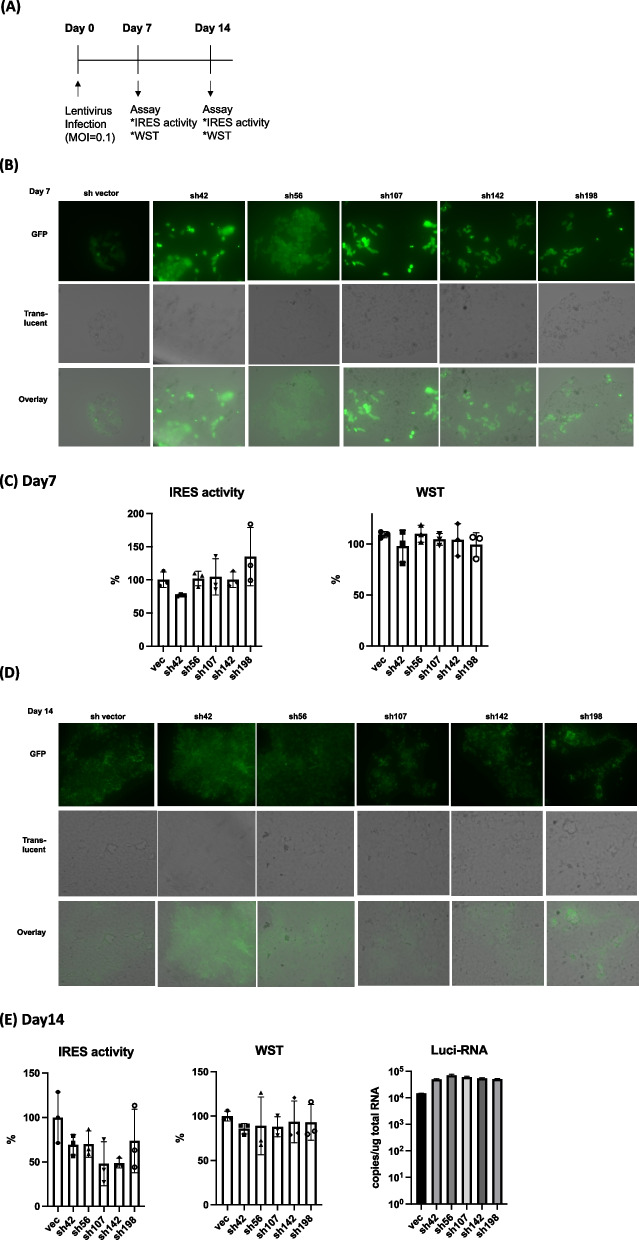
Comparison of shRNA-expressing lentivirus infection with CSFV-IRES activity. **A** Flow chart of lentivirus infection and assay of IRES and WST. **B** After 7 days of lentivirus infection, lentivirus vectors were detected under a fluorescent microscope Bz- × 700 (× 200). Translucent and merged images are shown. **C** IRES activity (left), and WST (right) after 7 days of lentivirus infection. Percentages of IRES activity in cells with or without siRNA treatment are indicated. *P*-value < 0.05 compared with control are indicated (One-way ANOVA and Dunnett’s multiple comparison test). **D** After 14 days of lentivirus infection, lentivirus vectors were detected under a fluorescent microscope Bz- × 700 (× 200). Translucent and merged images are shown. **E** IRES activity (left) and WST (middle) after 7 days of lentivirus infection. The percentages of IRES activity of vector control and siRNA-treated cells are indicated. One-way ANOVA was performed, and a *P*-value < 0.05 compared with the control is indicated (One-way ANOVA and Dunnett’s multiple comparison test). The amount of luciferase RNA in shRNA-expressing lentivirus-infected cells was measured by qRT-PCR (right). Vertical bars indicate the standard deviations. Representative results of three experiments are shown

**Fig. 4:**
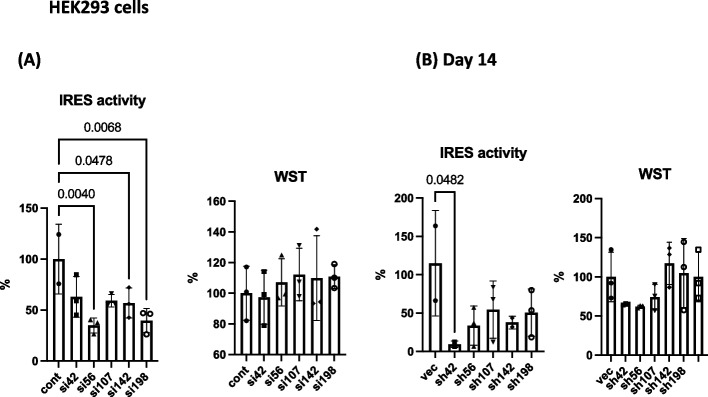
Efficacy of siRNA and shRNA in HEK293 cells. **A** Percentage of IRES activity in control and siRNA-treated pCI5 cells (left). Cell viability was measured using the WST assay and indicated by percentage to the OD_450_ value of control siRNA treated cells (right). Assay was performed after 72 h. Vertical bars indicate the standard deviations. One-way ANOVA and Dunnett’s multiple comparison test were conducted for all samples. *P*-value < 0.05 is indicated. **B** IRES activity (left), and WST (right) after 14 days of lentivirus infection to pCI5 cells. Percentages of IRES activity in cells with or without siRNA treatment are indicated. *P*-value < 0.05 compared with control are indicated (One-way ANOVA and Dunnett’s multiple comparison test)

Next, we constructed shRNA-expressing vectors using the PLL3.7 plasmid, as described in Materials and Methods. Using these vectors, we generated lentiviruses expressing shRNA (Table 2). After infection with lentivirus (MOI = 0.1), lentivirus vectors were detected on day 7 (Fig. 3A to C, left) without significant cytotoxicity (Fig. 3C, right). The presence of lentivirus was confirmed by the detection of GFP in the pLL3.7 vector [18] (Fig. 3B). After 14 days, lentivirus vectors were detected (Fig. 3D) and CSFV-IRES activity was suppressed by sh42, sh56, sh107, and sh142 (Fig. 3E, left), without significant cytotoxicity (Fig. 3E, middle). The levels of the luciferase RNA gene were similar among shRNA treated cells (Fig. 3E, right). The effect of siRNAs and shRNAs to CSFV-IRES was examined in CSFV-IRES expressing HEK293 cells (pCI5) [15] (Fig. 4) and significant effect was observed in si56, si142, si198 (Fig. 4A), and sh42 (Fig. 4B) treated cells.

**Table 2:**
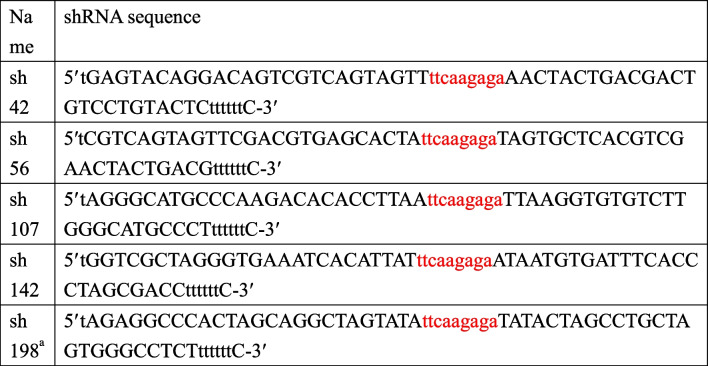
shRNA vector sequences targeting CSFV-IRES

^a^red characters indicate loop sequence

### Evaluation of the effect of siRNA and shRNA on CSFV replicon RNA

**Fig. 5:**
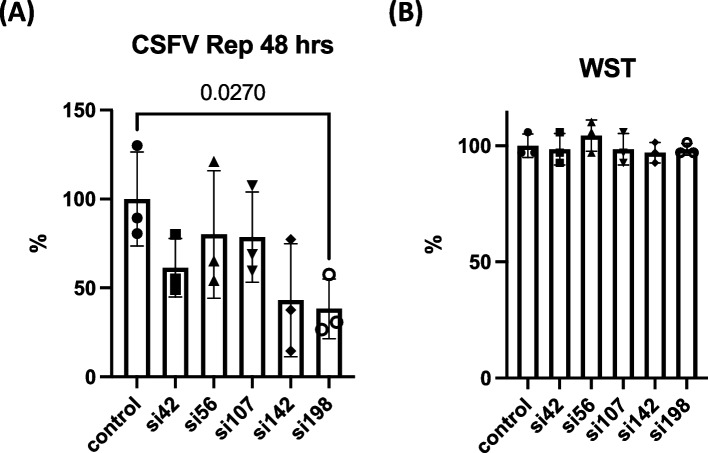
Comparison of siRNA with the CSFV replication activity. **A** The percentages of luciferase activity in control and siRNA-treated cells are indicated. *P*-value < 0.05 compared with control is indicated (One-way ANOVA and Dunnett’s multiple comparison test). **B** Cell viability was measured using the WST assay and indicated by the OD_450_ value. Vertical bars indicate the standard deviations. Representative results of three experiments are shown

The efficacy of siRNA against CSFV replicon RNA was examined using rGPE-Npro-Luc-IRES-NS3 RNA [19] (Fig. 5). After 48 h of siRNA transfection, CSFV replicon RNA was transduced by electroporation, and replication activity was measured by luciferase activity, which can reflect the CSFV replication activity within 24–48 h [19]. As a result, si198 showed a significant decrease (38.3% of control) in luciferase activity (Fig. 5A) without cytotoxicity (Fig. 5B).

**Fig. 6:**
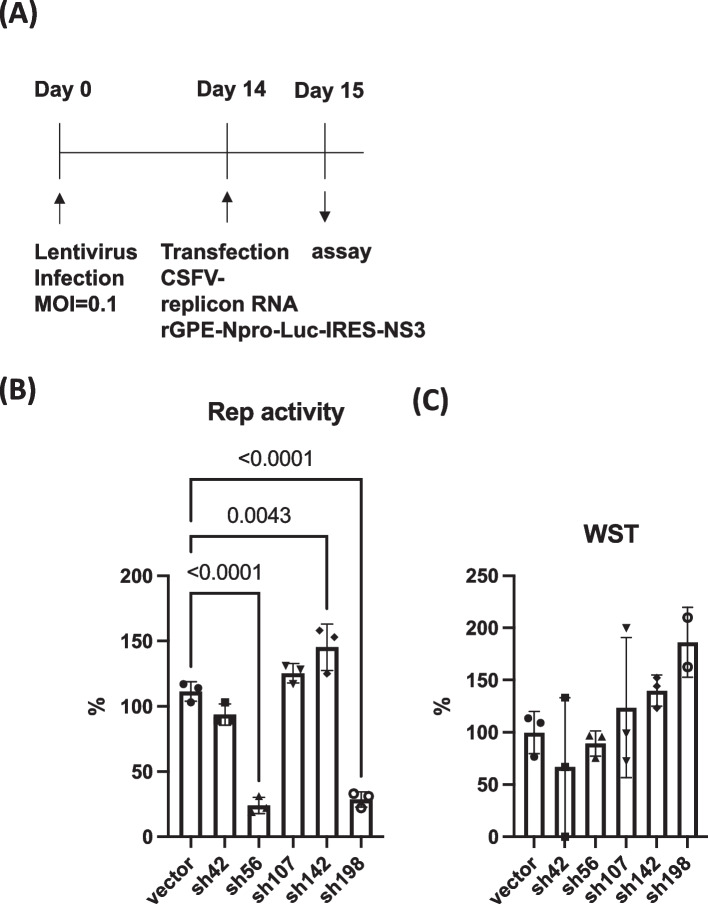
Comparison of shRNA-expressing lentivirus infection with CSFV replication activity. **A** Flow chart of lentivirus infection and assay of CSFV replication using CSFV replicon (rGPE-Npro-Luc-IRES-NS3). **B** Percentages of replication (luciferase) activity in control and cells infected with lentivirus are indicated. *P*-value < 0.05 compared with control is indicated (One-way ANOVA and Dunnett’s multiple comparison test). **C** Cell viability was measured using the WST assay and indicated by the OD_450_ value. Vertical bars indicate the standard deviations. Representative results of three experiments are shown

To examine the efficacy of shRNA in CSFV replicon RNA, we infected the lentivirus vector-expressing shRNA at a multiplicity of infection of 0.1 and incubated for 14 days (Fig. 6 A). We then transduced the CSFV replicon (rGPE-Npro-Luc-IRES-NS3 [19]) RNA into cells by electroporation, as described in the Methods section. Significant suppression of luciferase activity was observed following treatment with sh42, sh56, and sh198 lentiviruses (93.7%, 24%, and 28.7% of control, respectively) (Fig. 6B) without significant cytotoxicity (Fig. 6C).

**Fig. 7:**
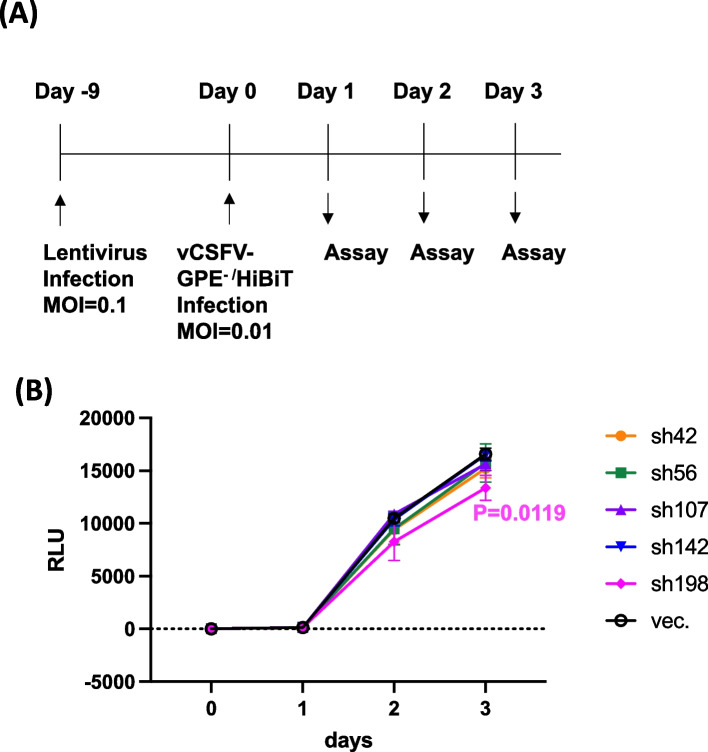
Effect of shRNA-expressing lentivirus infection to CSFV infection. **A** Flow chart of lentivirus infection and assay of CSFV infection using CSFV clone (vCSFV GPE.^−^/HiBiT). **B** Effect of shRNA expressing lentivirus to CSFV infection measured by HiBiT. Days indicate the timing after CSFV infection. Statistical analyses by one way ANOVA and Dunnett’s multiple comparison test were performed, and significant suppression by sh198 at day3 was calculated (*p* = 0.019)

The effect of shRNA on CSFV infection was examined using vCSFV GPE^−^/HiBiT, as described in Materials and Methods. SK-L cells were infected with lentivirus (day 0) at MOI = 0.1 and infected with vCSFV GPE^−^/HiBiT [20] after 9 days (Fig. 7A). Virus amount was measured by count of HiBiT (Fig. 7B). As a result, significant decrease of virus amount was observed by sh198 after 3 days post infection when compared with vector control (Fig. 7).

## Discussion

The results of this study demonstrated the effect of RNA interference by siRNA and shRNA targeting the domain III region of CSFV-IRES. All designed si/shRNA significantly inhibited IRES activity in the bi-cistronic vector or CSFV replicon RNA. The si42 targeting domain IIIa, si107 (domain IIIc), and si198 targeting domain IIIf showed significant suppression of IRES activity after 72 h of treatment without significant change in the amount of luciferase RNA. This may indicate that the siRNA specifically targets the IRES region to suppress translation. Consistent with this observation, domains IIIa, IIIb, and IIIc have been reported to support eIF3 binding [16, 21]. Some residues within domains IIIa, IIIc, IIId_1_, IIIe, and IIIf interact with ribosomal proteins [17], suggesting their roles in IRES function. Domain IIIf, together with domain IIId, has been reported to have a significant role in the formation of the 43S scaffold for the 80S ribosome formation and subsequent translation initiation [22, 23].

Longer expression (14 days) of shRNA using the lentivirus vector showed the suppressive effect to IRES activity. This may indicate that shRNA-expressing lentivirus vectors showed their efficacy over 14 days, which may provide insight for sustained suppression of CSFV. In fact, expression of sh56 (domain IIIb) can significantly suppress CSFV-IRES activity in CSFV replicon RNA, and sh198 significantly suppressed IRES activity in CSFV replicon RNA after 14 days of treatment and CSFV infection after 9 days of treatment. This may indicate the possibility that sh198 expressing lentivirus can be applicable for establishment of the CSFV resistant animal. The CSFV-IRES is composed of stem-loop structures like the hepatitis C virus (HCV)-IRES [21, 24], which may complicate an RNA silencing strategy. However, the IRES in the 5′UTR might be an attractive target region for RNA silencing in vivo [25], as it is highly conserved among virus serotypes [18, 26].

## Conclusion

The results of this study indicate that after 14 days of infection with lentiviral shRNA, suppression of CSFV-IRES activity occurred in both di-cistronic RNA and CSFV infection by sh198. Therefore, this shRNA could be used to establish CSFV-resistant transgenic pigs, as reported previously [11]. Future studies are required to establish transgenic animals expressing sh198 and examine their resistance to CSFV infection. CSFV-resistant pigs could become a powerful tool for the prevention and control of CSFV epidemics, as wild animals such as wild boars are carriers of CSFV and make regulation difficult [27, 28].

## Methods

### Cells

Swine kidney line L (SK-L) cells were originally established by Japanese researchers [29] and cultured, as described previously [15]. The vector containing CSFV-IRES [30] was a gift from Professor Graham J. Belsham of the University of Copenhagen. The pCAGGS-Neo vector was constructed as described by Ide et al. [15], and the CSFV-IRES cDNA (nt. 124–401) was excised from a reporter plasmid [30] using EcoRI and NcoI and inserted between the *Renilla* and firefly luciferase genes. The HEK293 cells were originally obtained from the American Type Culture Collection (ATCC) [31] and those expressing pCAGGS-Neo-CSFV-IRES (clone pCI5) were established as previously described [18]. Further, DNA sequencing was performed by Eurofins Genomics Co. (Tokyo, Japan), and DNA sequence characterization was performed using GENETYX-Mac software (GENETYX Co., Tokyo, Japan) and GENBANK. Cell viability was evaluated using tetrazolium salt (WST)-1 cell proliferation assays (TAKARA Bio Inc.) by determining the optical density at 450 nm (OD_450_), according to the manufacturer’s instructions. Luciferase assays were performed using a Dual-Luciferase Reporter Assay System (Promega, Madison, WI, USA). Luminescence was measured using EnVision (Perkin Elmer Co.) [31].

### siRNA transfection

siRNAs targeting CSFV-IRES (Table 1) were designed using the BLOCK-iT RNAi Designer (Thermo Fisher Scientific, Waltham, MA, USA), and an ON-target plus siRNA control (Horizon/Dharmacon, Lafayette, CO, USA) was used as a control [15]. Subsequently, siRNA (5 nM) reverse transfection was performed using Lipofectamine RNAiMAX reagent (Invitrogen), as described previously [15]. After 48–72 h, assay was performed.

### Transfection, plasmid construction, and lentiviral infection

Plasmid transfection was performed using Lipofectamine LTX reagent (Invitrogen, Carlsbad, CA, USA) according to the manufacturer’s instructions after the cells reached 50–70% confluence. For cell line establishment, SK-L cells were cultured in a medium containing G418 (300 μg/mL) after transfection with the pCAGGS-Neo/CSFV-IRES vector. After 3–4 weeks, the G418-resistant cells were identified as colonies. Subsequently, siRNA (5 nM) reverse transfection was performed using Lipofectamine RNAiMAX reagent (Invitrogen) according to the manufacturer’s instructions. The CSFV-IRES shRNA expression vectors were constructed using the pLL3.7 vector (cat. no. 11795; Addgene, Watertown, MA, USA). The shRNA sequences are listed in Table 2; they were subcloned under the U6 promoter in the pLL3.7 vector. Lentivirus vectors were packaged using MISSION Lentiviral Packaging Mix (Sigma-Aldrich, St. Louis, MO, USA), and cells were infected with lentivirus according to the manufacturer’s instructions. Titration of lentivirus was performed by detecting green fluorescent protein (GFP) using a fluorescence microscope (Bz- × 700; Keyence, Osaka, Japan). CSFV replicon (rGPE-Npro-Luc-IRES-NS3) RNA was synthesized and transfected into SK-L cells by electroporation, as described [19]. The vCSFV GPE^−^/HiBiT recombinant classical swine fever virus encoding the HiBit luciferase gene [20] was infected to shRNA vector transduced SK-L cells, as described [32].

### Quantitation of the luciferase gene

The amount of luciferase mRNA in pCI5 cells with or without siRNA or shRNA treatment was measured in isolated total RNA using the RNAeasy mini kit (QIAGEN) with DNase I treatment. Luciferase mRNA was measured using qRT-PCR Brilliant III SYBR master mix (Agilent) and pGL3basic 1098S primer (5’-CAAGGATATGGGCTCACTGA-3’) and 1348R primer (5’- CAGAATGTAGCCATCCATCC-3’) using the CFX Connect real-time PCR analysis system (BioRad).

### Statistical analysis

All data are presented as mean ± standard deviation (S.D.) from three independent experiments, and figures were generated using GraphPad PRISM (version 9) software. Statistical analysis was first performed by one-way ANOVA, followed by the Dunnett’s multiple comparison test to evaluate significant differences. The results with *P*-values < 0.05 were considered statistically significant.

### Supplementary Information

**Additional file 1. **

## Data Availability

All the data and materials in this study can be provided on reasonable request.
