# Supplementary material for: Development of short hairpin RNA expression vectors targeting the internal ribosomal entry site of the classical swine fever virus genomic RNA
Source: BMC Biotechnol. 2023 Sep 8;23:37. doi: 10.1186/s12896-023-00805-6 (PMC10492304; doi:10.1186/s12896-023-00805-6)
Supplement: Supplementary file 1 — Additional file 1. [file 12896_2023_805_MOESM1_ESM.docx]

Additional file

**Development of short hairpin RNA expression vectors targeting the internal ribosomal entry site of the classical swine fever virus genomic RNA**

Riai Okamoto^1^, Nobumasa Ito^1^, Yutaro Ide^1^, Bouchra Kitab^1^, Yoshihiro Sakoda^2^, and Kyoko Tsukiyama-Kohara^1,3*^

^1^Transboundary Animal Disease Center, Joint Faculty of Veterinary Medicine, Kagoshima University, Kagoshima 890-0065, Japan

^2^Department of Disease Control, Faculty of Veterinary Medicine, Hokkaido University, Hokkaido 060-0818, Japan

^3^Laboratory of Animal Hygiene, Joint Faculty of Veterinary Medicine, Kagoshima University, Kagoshima 890-0065, Japan

^4^Correspondence: kkohara@vet.kagoshima-u.ac.jp; Tel.:81-99-285-3589

**Figure S1.** Establishment flow of bicistronic CSFV-internal ribosomal entry site (CSFV-IRES)-expressing SK-L or HEK293 cells. A bicistronic reporter construct was designed to contain the CSFV-IRES element located between the *Renilla* (R-luc) and firefly luciferase (F-luc) genes. The bicistronic reporter gene was excised using the restriction enzymes EcoRV and BamHI and ligated into the pCAGGS-Neo/MCS vector digested with EcoRV and BamHI. Transfected cells were selected with G418.
